# Differences in the Plastispheres of Biodegradable and Non-biodegradable Plastics: A Mini Review

**DOI:** 10.3389/fmicb.2022.849147

**Published:** 2022-04-25

**Authors:** Chu Peng, Jiao Wang, Xianhua Liu, Lei Wang

**Affiliations:** ^1^MOE Key Laboratory of Pollution Processes and Environmental Criteria, College of Environmental Science and Engineering, Nankai University, Tianjin, China; ^2^College of Environmental Science and Engineering, Tianjin University, Tianjin, China

**Keywords:** microbial community, driving force, health risk, biodegradable plastic, biofilm

## Abstract

There has been a steady rise in the production and disposal of biodegradable plastics. Unlike the microorganisms present in the biofilms on non-biodegradable plastic surfaces (the “plastisphere”), the plastisphere of biodegradable plastic has not been well-characterized. As the polymer structure of biodegradable plastic has a higher microbial affinity than that of non-biodegradable plastic, their plastispheres are assumed to be different. This review summarizes the reported differences in microbial communities on the surface of biodegradable and non-biodegradable plastics, discusses the driving forces behind these differences, and discusses the potential environmental risks. Overall, the plastisphere biomass on the surface of non-biodegradable plastic was observed to be lower than that of biodegradable plastic. The community structure of microbes in both plastispheres was diverse, mainly due to the properties of the plastic surface, such as surface charge, hydrophilicity/hydrophobicity, roughness, and bioavailability of polymer components for microbes. Further research should focus on developing biodegradable plastic that degrade faster in the environment, revealing the mechanism of enrichment of ARGs and potential pathogens on plastics, and understanding the potential influence of plastispheres on the evolution and selection of plastic-degrading microbial potential.

## Introduction

Since the 1950s, global production of 8,300 million metric tons (Mt) of plastics have been produced (Geyer et al., 2017). Plastic polymers are extremely versatile and durable, with non-biodegradable plastics being the most widely used plastic polymers. However, the continuous rise in plastic waste has prompted a major environmental problem that has overshadowed the importance of plastics in the global economy [Organization for Economic Cooperation and Development (OECD), 2021]. It is estimated that 79% of plastic waste makes its way to landfills or oceans, impacting aquatic and terrestrial ecosystems (Geyer et al., 2017). These plastics persist in these environments for a significant period of time and contribute to the accumulation of plastics and microplastics (MPs; Alimi et al., 2018; Boots et al., 2019; Kumar et al., 2021).

Plastic polymers can be degraded by abiotic factors (weathering, fragmentation, and photooxidation) and biotic factors (microorganisms and enzymes; Haider et al., 2019; Ju et al., 2021). Biodegradable plastics are considered environmentally friendly alternative materials to alleviate the increasing plastic waste and have been extensively used in recent years, such as polylactic acid (PLA), poly(butyleneadipate-co-terephthalate; PBAT), poly(butylene succinate; PBS), and polyhydroxyalkanoate (PHA; Supplementary Table S1).

Microorganisms colonize the surface of plastics to form a biofilm, which is called the “plastisphere” (Zettler et al., 2013). The microbial community of the plastisphere is distinct from that of the surrounding water, soil, or natural solid matrix (De Tender et al., 2015; Wu et al., 2019; Zhang et al., 2019). Plastisphere related detrimental ecological effects have been discovered continuously in past years. It has been established that MPs can act as a reservoir and refuge for antibiotic resistance genes (ARGs) and potential pathogens (Schmidt et al., 2014; Foulon et al., 2016; De Tender et al., 2017; Yang et al., 2019). Moreover, they are easily eaten by animals, found in human food, and inhaled through dust, thereby posing a health risk to living beings (Van Franeker et al., 2011; Van Cauwenberghe and Janssen, 2014; Zhang et al., 2020).

The degradation of biodegradable plastics can still require long time scales, even though being typically more effective than for non-biodegradable plastics. Kamiya et al. (2007) observed that a PLA film was not degraded even after 120 d in soil. Moreover, PHA lost merely 7.9% after 450 d of composting (Mercier et al., 2017). Given that the polymeric structure of biodegradable plastic endows high microbial affinity, its microbial colonization capacity is assumed to be different from that of non-biodegradable plastic. Through field investigation and laboratory studies, many researchers have studied the microbial community on biodegradable surfaces in different environments (Mercier et al., 2017; Dussud et al., 2018; Gonzalez-Pleiter et al., 2021). However, there is still a lack of understanding of the characteristics of and the differences between biodegradable and non-biodegradable plastispheres.

**Figure 1:**
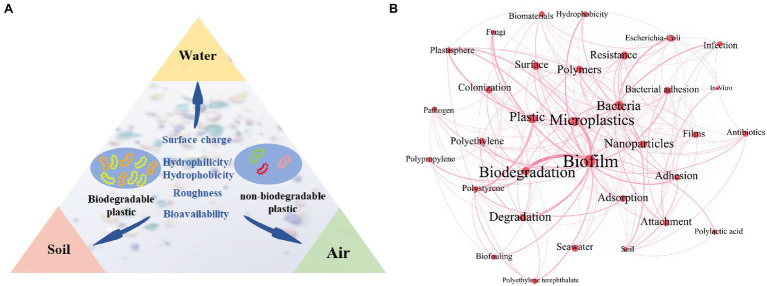
Microbial differences in the biodegradable plastisphere and non-degradable plastisphere in three environments (air, water, and soil; **A**). Co-occurrence network analysis of plastic- and biofilm-related keywords in current studies **(B)**. Each keyword on the map is displayed as a node, with size determined by the occurrence. Keyword relationships are shown as edges of varying thickness determined by the co-occurrence.

This review aims to summarize the differences in microbial communities on the surface of biodegradable and non-biodegradable plastics in various environments, discuss the driving forces of community differences based on the characteristics of the plastics, and delineate the potential health risks of biodegradable plastic biofilms in the environment (Figure 1A).

## Production, Emission, and Environmental Distribution

In 2019, the global production of plastics reached 368 million Mt., with polyethylene (PE, 29.8%), polypropylene (PP, 19.4%), and polyvinyl chloride (PVC, 10%) being the dominant polymers (Europe Plastics, 2020). However, owing to the COVID-19 pandemic, plastic production declined in 2020. For example, production across the EU is expected to fall by 8.5% in 2020 (Europe Plastics, 2020). On the other hand, despite the impact of the COVID-19 pandemic, the production of biodegradable plastics in 2020 was 2.11 million Mt., which is a growth of 8.2% (Europe Bioplastics, 2021).

To the best of our knowledge, no research has quantified the recycling rate of degradable and non-biodegradable plastics. Biodegradable plastics are mainly used as a replacement for single-use plastic (shopping bags, beverage straws, food containers, etc.) and are believed to be more environmentally friendly (Rai et al., 2021). Thus, their release into the environment has increased. MPs with sizes less than 5 mm are a hot topic in the current environmental research (Figure 1B). MPs are ubiquitous, found in urban dust as well as the deep-sea sediment cores of Antarctica (Liu et al., 2019; Cunningham et al., 2020). Since the production of non-biodegradable plastics is significantly higher than that of biodegradable plastics, most MPs that are currently detected are non-biodegradable. Few studies have focused on biodegradable MPs. For example, Peng et al. (2021) studied PLA particles in indoor dust, Wei et al. (2021) explored PBAT in freshwater and seawater, and Cai et al. (2018) researched polycaprolactone (PCL) in oceans.

## Comparison of the Microbial Community Colonizing Biodegradable and Non-biodegradable Plastic

In the last decade, microbial communities on the surface of non-biodegradable plastics have been extensively investigated. Few studies that have addressed biodegradable plastic to date (Table 1) concluded that non-biodegradable plastics have lower biomass than biodegradable plastic. Dussud et al. (2018) incubated biodegradable poly(3-hydroxybutyrate-co-3-hydroxyvalerate; PHBV) and PE in seawater for 6 weeks and concluded that the cell content on the PHBV surface was five times higher than that on the PE surface. A similar result was obtained for indoor air where PLA particles presented higher 16S rRNA gene counts and microbial biomass than PE and PP after 30 d (Peng et al., 2021). In an outdoor composting experiment, the microbial colonization (represented by DNA quantities) on polyethylene terephthalate (PET) biofilms was found to be lesser than that on PHA and PBS biofilms (Mercier et al., 2017). All of these studies suggest that biodegradable plastics favor higher microbial colonization.

**Table 1:** The plastisphere compared with biodegradable and non-biodegradable plastics.

Polymer types	Exposure environment	Abundant microbes		References
		Biodegradable plastic	Non-biodegradable plastic	
PBAT, PLA; PET, PS, PVC	Seawater	Bacterial community: *Vibrionaceae*, *Clostridiaceae*, *Actinomycetaceae*, and *Flavobacteriaceae*	Bacterial community: *Vibrionaceae*, *Clostridiaceae*, *Actinomycetaceae*, and *Flavobacteriaceae*	Delacuvellerie et al., 2021
PLA; PP, PE	Seawater	Bacterial community: unidentified Cyanobacteria, unidentified Alphaproteobacteria, and unidentified Gammaproteobacteria	Bacterial community: unidentified Cyanobacteria, unidentified Alphaproteobacteria, and unidentified Gammaproteobacteria	Zhang et al., 2021a
PHBV; PE	Seawater	Bacterial community: *Neptiniibacter, Phaeobacter,* and *Roseobacter*	Bacterial community: *Solimonas*, *Thalassolituus*, and *Alcanivorax*	Dussud et al., 2018
PLA; PS	Seawater	Bacterial community: Planctomycetaceae and Flavobacteriales	Bacterial community: Saprospiraceae and Planctomycetaceae	Odobel et al., 2021
PBS; PP	Seawater	Bacterial community: *Maribius*, *Neptuniibacter*, and *Bacillus*	Bacterial community: *Polycyclovorans* and *Marivita*	Jacquin et al., 2021
PHB; PE	Freshwater	Bacterial community: *Moraxellaceae*_unclassified, *Rhodoferax*, *Comamonadaceae*_unclassified, *Polaromonas*, and *Erythromicrobium*; Fungal community: *Betamyces*, *Arrhenia,* and *Malassezia*	Bacterial community: *Erythromicrobium*, *Rhodobacter*, Comamonadaceae_unclassified Fungal community: *Betamyces*, *Cryptococcus*, *Paranamyces*, and *Xylodon*	Gonzalez-Pleiter et al., 2021
PBAT; PET	Soil	Bacterial community: Comamonadaceae, Bradyrhizobiaceae, Caulobacteraceae Fungal community: Davidiellaceae, Leptosphaeriaceae, Shiraiaceae	Bacterial community: Clostridiaceae, Hyphomicrobiaceae, Methylococcaceae Fungal community: Monoblepharidaceae, Mortierellaceae, Glomeraceae	Mercier et al., 2017
PLA; PE	Soil	Bacterial community: *Burkholderia*, *Bosea*, and *Bradyrhizobium* Fungal community: *Penicillium*, *Talaromyces*, and *Westerdykella*	Bacterial community: *Mycobacterium*, *Chitinophaga*, and *Nocardia* Fungal community: *Penicillium* and *Fusarium,*	Zhang et al., 2021b
PLA; PBAT PE	Soil	Bacterial community: *Saccharimonadales*, *Propionibacteriales*, and *Rhizobiales*	Bacterial community: *Propionibacteriales*, *Pyrinomonadales*, and *Chthoniobacterales*	Rüthi et al., 2020
PLA/PHA; PE	Soil	Bacterial community: *Methylobacterium*, *Arthrobacter*, and *Sphingomonas*	Bacterial community: *Conthreep*	Bandopadhyay et al., 2020
PLA; PE, PP	Indoor dust	Bacterial community: *Ralstonia*, *Corynebacterium*, and *Sphingomonas*	Bacterial community: *Ralstonia*, *Paracoccus*, and *Deinococcus*	Peng et al., 2021

Furthermore, the microbial diversity between biodegradable and non-biodegradable plastics is distinct, as indicated by the Simpson and Shannon indices. Mercier et al. (2017) investigated the bacterial richness and diversity indices among plastic biofilms and observed higher bacterial operational taxonomic units (OTUs) and Shannon index on the PET surface, as compared to the three biodegradable polymers. Another study compared biodegradable and non-biodegradable plastic mulch films, and observed that the PLA/PHA film was colonized by a microbial community with remarkably fewer bacterial OTUs and a lower Simpson index than PE plastic (Bandopadhyay et al., 2020); this indicated that hydrophobic and non-biodegradable surfaces selected microorganisms less discriminately. Furthermore, a study on the fungal communities on the PET surface revealed that the number of OTUs and the Shannon index was nearly twice that of the BAT, PBS, and PHA surface (Mercier et al., 2017). Therefore, non-biodegradable plastics attract more diverse bacteria and fungi than biodegradable plastics.

The bacterial and fungal communities of biodegradable and non-biodegradable plastispheres are different in aquatic and terrestrial environments. Numerous studies have investigated biofilm colonization on biodegradable and non-biodegradable plastic surfaces in marine environments. Eich et al. (2015) found distinct biofilm communities on two polymer-type surfaces at benthic and pelagic sites. Diatoms of *Striatella* sp. were more abundant on biodegradable plastic than on PE plastic after 33 d (Eich et al., 2015). Following seawater incubation for 15 months, microbial communities on PLA were significantly different from that of seven other synthetic substrates, with *Leptobacterium* being the major contributor to the dissimilarity (Kirstein et al., 2018). Odobel et al. (2021) reported on the variations in mature biofilms on different polymer surfaces. They observed that Planctomycetaceae (12%) was the most abundant in PLA, whereas Saprospiraceae (19%) was the most abundant in polystyrene (PS; Odobel et al., 2021). Another study that focused on the freshwater environment concluded that the microbial composition between polyhydroxybutyrate (PHB) and PE varied remarkably, and the PHB surface consisted primarily of unclassified Moraxellaceae, whereas the PE surface hosted a significant amount of *Erythromicrobium* spp. (Gonzalez-Pleiter et al., 2021).

An increasing number of studies have explored terrestrial plastispheres and indicated that the plastispheres of biodegradable and non-biodegradable plastics in soil environments are distinct. By burying multiple plastics in two different soils, Rüthi et al. (2020) observed that the PBAT plastisphere was enriched with *Collimonas* and *Pseudomonas*, whereas a larger number of *Aeromicrobium* and *Nocardioides* were found on the PE surface. At the genus level, the plastisphere on biodegradable plastic showed a significantly greater abundance of *Methylobacterium*, *Arthrobacter*, and *Sphingomonas*; on the other hand, PE mulch had a higher amount of Ciliophora within the CONthreeP (Bandopadhyay et al., 2020). With respect to the fungal community, PLA/PHA and PE plastic films had similar eukaryotic compositions but different proportions of taxa distribution at the class level (Bandopadhyay et al., 2020). Another report suggested that *Oidiodendron* and *Alternaria* were enriched in the PLA plastisphere, whereas *Phlebia* was higher in the PE plastisphere (Rüthi et al., 2020). Thus, in varying environments, different plastisphere microbiomes harbor distinct biomass, microbial diversity, and community composition in biodegradable and non-biodegradable plastics.

## Analysis of Driving Forces That Cause Microbial Community Differences

Biofilm formation can be divided into five stages: the initial adhesion of cells, secretion of extracellular polymeric substances, biofilm growth and community formation, biofilm structure maturation, and cell detachment and dissipation (Davey and O'Toole, 2000; Stoodley et al., 2002; Wright et al., 2020). The impact of polymeric properties on the microbial community of the surface biofilm at different stages should be considered based on the four properties of polymers: surface charge, hydrophilicity/hydrophobicity, roughness, and bioavailability.

Electrostatic interactions play an important role in the adsorption of microorganisms on the surface of the material, particularly during the initial cell adsorption stage. The point of zero charge (PZC) of most bacterial cells ranges between 3 and 4, leading to a negative charge on bacterial cells in most environments (Soni et al., 2008). This indicates that materials with positively charged surfaces facilitate the adsorption of bacteria. Terada et al. (2012) modified the PE surface and found that the cell adhesion density of *Escherichia coli* on a positively charged surface was 23 times higher than that on a negatively charged surface; moreover, the biofilm on the surface of the positively charged material was denser and more homogeneous (Terada et al., 2012). Different PZCs on the surfaces of plastics, such as PLA (9.95) and PP (4.26; Tavolaro et al., 2016; Xu et al., 2018) can lead to varying adsorption interactions of microorganisms.

Other factors that affect the adhesion behavior of microorganisms include surface hydrophilicity and hydrophobicity of plastics. A previous study found that the adhesion of nine *Staphylococcus epidermidis* strains was stronger on the surface of PP with strong hydrophobicity than that on glass (Cerca et al., 2005). Similarly, a significant amount of *P. aeruginosa* PAO1 was found on the surface of materials with a larger water contact angle (more hydrophobic) in a fluid medium (Lee et al., 2010). Generally, surfaces with higher N/C values are more hydrophobic and those with higher O/C values are more hydrophilic; this leads to differences in the communities on plastic surfaces (Desrousseaux et al., 2013). Differences in the water contact angles on plastics, such as PET (86.2), PP (99), PLA (77), and PBAT (78.6; Thanki et al., 2006; Pandiyaraj et al., 2008; Huang et al., 2020) may lead to different microorganism colonization characteristics on their surfaces.

Plastic surfaces are not smooth; this is especially true for plastic wastes that accumulate in the environment. Biodegradable plastics that enter the environment tend to have rougher surfaces, which provide a larger specific surface area for microbial colonization. Purahong et al. (2021) observed that on burying a poly(butylene succinate-*co*-adipate; PBSA) film in the soil, its smooth morphology got corroded and became rough after 180 d. Conversely, Jacquin et al. (2021) found that there was no clear morphological change on the surface of PP after incubating it in seawater for 6 weeks. On the other hand, the surfaces of PHBV and PBAT got significantly corroded in seawater (Jacquin et al., 2021).

Biodegradability tends to affect the microbial communities on polymers. More microorganisms can use biodegradable plastics as their sole carbon source. The degradation of PLA, which is the most productive and studied biodegradable plastic (Figure 1B), has been linked to 28 strains of 13 genera of bacteria and 4 genera of fungi (Qi et al., 2017). In comparison, fewer microorganisms can degrade non-biodegradable plastic, such as PET, PP, and PA (Danso et al., 2019). During their environmental degradation, biodegradable plastics, such as PLA and PCL, release small molecules (lactic acid and caprolactone), which are readily utilized by environmental microorganisms as carbon and energy sources (Hakkarainen, 2001). Thus, microorganisms that can use biodegradable plastics are at an advantage, resulting in higher abundances. In a previous study, 13 genera of PLA-degrading bacteria were identified on the surface of PLA; of these, 11 genera were found to be more abundant on the surface of PLA than on the surface of other plastics in the same indoor environment (Peng et al., 2021). Notably different bacterial communities were observed on the surfaces of biodegraded PBAT and non-biodegraded PBAT in soil, and several abundant members of *Bradyrhizobium*, *Ramlibacter*, and *Variovorax* were identified as novel potential degraders (Han et al., 2021). However, the actual biodegradation ability of microorganisms that are remarkably abundant on biodegradable plastics is rarely verified.

## Health Effects Related to Biodegradable Plastics

Currently, health threats of plastisphere are mainly related to two aspects: pathogenic bacteria on the plastic surface and ARGs carried by the microorganisms. However, few researchers have investigated the potential threats induced by biodegradable plastics.

Several studies have reported that potentially pathogenic organisms are present on marine plastic debris, the most abundant being *Vibrio* spp. (Schmidt et al., 2014; Kirstein et al., 2016). *Vibrionaceae* and *Pseudoalteromonadaceae* were commonly detected on the surfaces of MPs but rarely observed in seawater and sediment communities (De Tender et al., 2017). Two opportunistic human pathogens (*Pseudomonas monteilii* and *Pseudomonas mendocina*) and one plant pathogen (*Pseudomonas syringae*) were detected on a PVC biofilm, but not on biofilms formed on natural substrates, such as rock and leaf (Wu et al., 2019). A range of potential pathogens was detected in the plastisphere of the soil environment, with more potential pathogens present on the surface of MPs (PVC, polyamide, PE, and PS) than in the soil (Zhu et al., 2022). Another study revealed that MPs in the soil served as selective artificial microhabitats that not only attracted distinct fungal communities but also accumulated opportunistic human pathogens, such as cryptococcal and Phoma-like species (Gkoutselis et al., 2021). A previous study on an indoor environment observed that larger biomass resulted in a higher potential pathogenic bacteria index on the surface of PLA particles, as compared to the surfaces of PP and PE particles (Peng et al., 2021). However, Oberbeckmann et al. (2017) found that the enrichment of potentially pathogenic bacteria on the surfaces of MPs and natural substrates did not vary significantly. Thus, additional research is required to determine whether a larger biomass on biodegradable plastic surfaces will promote the enrichment of pathogens.

The World Health Organization has declared ARGs to be the critical global public health risk of the century (Martinez et al., 2015). The selective affinity of ARGs for MPs has been widely reported in seawater (Yang et al., 2020), freshwater (Wang et al., 2020), soil (Zhu et al., 2022), and air environments (Peng et al., 2021). Yang et al. (2020) confirmed that microorganisms on the plastic surface showed higher antibiotic resistance than microorganisms in the surrounding water. Moreover, different enrichment capacities of ARGs on biodegradable plastics and non-biodegradable plastics have been demonstrated. In freshwater environments, the relative abundance of *ermB* (macrolides ARG) and *sulI* (sulfanilamide ARG) was significantly higher on the surface of PE than that of PHB (Gonzalez-Pleiter et al., 2021). On the contrary, the surface of PLA had a remarkably higher relative and absolute abundance of 18 ARGs, as compared to PP and PE in an air environment (Peng et al., 2021). Potential enrichment mechanisms of plastics with respect to ARGs are still unclear, and the health risks of biodegradable plastics and non-biodegradable plastics due to ARGs cannot be determined from current research.

## Conclusion and Future Perspectives

The extensive use of biodegradable plastics leads to an increase in their disposal in the environment. Although biodegradable plastic is endowed with better biodegradability than non-biodegradable plastic, it does not degrade quickly in the soil or water environment and continues to accumulate in the environment. Thus, future research should focus on developing plastics that degrade faster in the environment.

The microorganism community structures in the plastispheres of biodegradable and non-biodegradable plastics are distinct (Table 1) due to the differences in the intrinsic properties of plastics, such as surface charge, hydrophilicity/hydrophobicity, roughness, and bioavailability.

MPs can act as a reservoir and refuge for the selective enrichment of ARGs. Furthermore, larger biomass on the surface of biodegradable plastic augments health risks. The mechanism of this enrichment is still unknown, and the ability of plastics to enrich antibiotics may be one of the reasons, which needs further verification. A microenvironment with higher antibiotic concentration increases the survival and transfer of ARGs.

Microorganisms are still adapting to plastics. Numerous microorganisms, such as *I. sakaiensis* on PET (Yoshida et al., 2016), and their plastic-degrading enzymes, including protease and lipase, have been discovered; these enzymes catalyze the hydrolysis of PLA (Haider et al., 2019). Hence, the plastispheres of biodegradable and non-biodegradable plastics can evolve toward better utilization of the plastic substrates. Recognizing this evolution is important for the prediction of health and ecological threats.

## Author Contributions

CP and JW: data collection and analysis and writing–original draft preparation. XL and LW: writing-review and editing. All authors contributed to the article and approved the submitted version.

## Funding

This work was supported by the National Natural Science Foundation of China (41722304 and 42077336) and funded in part by the Tianjin Municipal Science and Technology Bureau (18JCJQJC47100), Key Project of Tianjin Natural Science Foundation (18JCZDJC33900), and the Ministry of Education, China (T2017002).

## Conflict of Interest

The authors declare that the research was conducted in the absence of any commercial or financial relationships that could be construed as a potential conflict of interest.

## Publisher’s Note

All claims expressed in this article are solely those of the authors and do not necessarily represent those of their affiliated organizations, or those of the publisher, the editors and the reviewers. Any product that may be evaluated in this article, or claim that may be made by its manufacturer, is not guaranteed or endorsed by the publisher.
