# Supplementary material for: Differences in the Plastispheres of Biodegradable and Non-biodegradable Plastics: A Mini Review
Source: Front Microbiol. 2022 Apr 25;13:849147. doi: 10.3389/fmicb.2022.849147 (PMC9082994; doi:10.3389/fmicb.2022.849147)
Supplement: Supplementary file 1 [file Table_1.DOCX]

**SUPPORTING INFORMATION**

**Differences in Plastispheres of Biodegradable Plastics and Non-biodegradable Plastics: A Mini Review**

**Chu Peng^#, 1^, Jiao Wang^#, 2^, Xianhua Liu^*, 2^, Lei Wang^*, 1^**

1. MOE Key Laboratory of Pollution Processes and Environmental Criteria, College of Environmental Science and Engineering, Nankai University, Tianjin 300350, China

2. College of Environmental Science and Engineering, Tianjin University, Tianjin 300354, China.

Table S1. Representative non-biodegradable and plastics biodegradable and their properties.

| Type | Polymer | [Abbreviation](javascript:;) | Density | Melting point | Usage |
| --- | --- | --- | --- | --- | --- |
| Non-biodegradable plastics | Low-Density Polyethylene | LDPE | 0.91-0.925 g/cm^3^ | 108-126℃ | Cling film,Milk carton lining |
|  | High-Density Polyethylene | HDPE | 0.945-0.965 g/cm^3^ | 142℃ | Food packaging, Shopping bags |
|  | Polypropylene | PP | 0.88-0.92 g/cm^3^ | 175℃ | Rigid packaging, technical parts |
|  | [Polystyrene](javascript:;) | PS | 1.07 g/cm^3^ | 105-110℃ | Packaging, consumer electronics, construction |
|  | Polyethylene terephthalate | PET | 1.36 g/cm^3^ | 255℃ | Bottles for toiletries, packing |
|  | [Polyamide](javascript:;) | PA | 1.13-1.15 g/cm^3^ | 215-260℃ | Textile filament, carpet filament, industrial filament |
|  | Polyvinyl chloride | PVC | 1.23 g/cm^3^ | 85℃ | Pipes, films and sheets, profiles and tubing |
| Biodegradable plastics | Poly(2-hydroxypropanoic acid) | PLA | 1.25-1.28g/cm^3^ | 140-152°C | Food packaging, bottles, cups and dishes and grocery bags |
|  | Poly(hydroxyalkanoate) | PHA | 1.25 g/cm^3^ | 175 °C | Wrap foods, coatings for paper and cardboard |
|  | Polycaprolactone | PCL | 1.021 g/cm^3^ | 60 °C | Food packaging and tissue engineering. |
|  | Poly(butylene succinate) | PBS | 1.25 g/cm^3^ | 114 °C | Packaging, agricultural film, and biomedical polymer materials |
|  | Poly(butylene adipate-  co-terephalate) | PBAT | 1.18-1.3 g/cm^3^ | 110-120℃ | Packaging and agricultural applications |

Reference:

Andrady, A.L. (2017) The plastic in microplastics: A review. *Mar. Pollut. Bull.* 119, 12-22.

Chong, S., Pan, G.T., Khalid, M., Yang, T., Hung, S., and Huang, C. (2017) Physical characterization and pre-assessment of recycled high-density polyethylene as 3D printing material. *J. Polym. Environ.* 25, 136–145.

Emblem, A. (2012) Plastics properties for packaging materials. *Packaging Technology*. 13, 287-309

Jiang, L., Zhang, J. (2017) Biodegradable and biobased polymers. *Applied Plastics Engineering Handbook*. 7, 127-143

Jordan, J.L. Casem, D. T. Bradley, J.M., Dwivedi, A.K., Brown, E.N., and Jordan, C.W. (2016) Mechanical properties of low density polyethylene. *J. Dynam. Behav. Mat.* 2, 411-420.

Li Z., Kong J., Ju D., Cao, Z., Han, L., and Dong, L. (2017) Thermal conductivity enhancement of poly(3-hydroxylbutyrate) composites by constructing segregated structure with the aid of poly(ethylene oxide). *Compos. Sci. Technol.* 149, 185-191.

Sharma, B.K., Moser, B.R., Vermillion, K.E., Doll, K.M., and Rajagopalan, N. (2014) Production, characterization and fuel properties of alternative diesel fuel from pyrolysis of waste plastic grocery bags. *Fuel Process. Technol.* 122, 79-90.

Sujaritjun, W., Uawongsuwan, P., Art, W.P., Hamada, H. (2013) Mechanical property of surface modified natural fiber reinforced PLA biocomposites. *Energy Procedia.* 34, 664-672.

<http://www.essentialchemicalindustry.org/polymers/polyesters>.

<https://www.okchem.com/news/0rL4VtXsa/Comparison-of-biodegradable-materials-PLA%2C-PBAT%2C-PHA-and-PBS>.

<https://plasticranger.com/pvc-melt-point-properties-applications-advantages-disadvantages>.
